# Adaptive traits of *Nitrosocosmicus* clade ammonia-oxidizing archaea

**DOI:** 10.1128/mbio.02169-24

**Published:** 2024-10-03

**Authors:** Saem Han, Seongwook Kim, Christopher J. Sedlacek, Adeel Farooq, Chihong Song, Sujin Lee, Shurong Liu, Nicolas Brüggemann, Lena Rohe, Miye Kwon, Sung-Keun Rhee, Man-Young Jung

**Affiliations:** 1 Interdisciplinary Graduate Program in Advance Convergence Technology and Science, Jeju National University, Jeju, South Korea; 2 Division of Microbial Ecology, Centre for Microbiology and Environmental System Science, University of Vienna, Vienna, Austria; 3 Department of Biology, University of Southern Indiana, Evansville, Indiana, USA; 4 Department of Biology Education, Jeju National University, Jeju, South Korea; 5 Core Research Facility, Pusan National University, Yangsan, South Korea; 6 School of Agriculture, Sun Yat-Sen University, Shenzhen, China; 7 Agrosphäre (IBG-3), Institut für Bio- und Geowissenschaften (IBG), Forschungszentrum Jülich GmbH, Jülich, Germany; 8 Thünen Institute of Climate-Smart Agriculture, Braunschweig, Germany; 9 Biodiversity Research Institute, Jeju Technopark, Jeju, South Korea; 10 Department of Microbiology, Chungbuk National University, Chungdae-ro,Seowon-Gu, Cheongju, South Korea; Korea Advanced Institute of Science and Technology, Daejeon, South Korea

**Keywords:** *Nitrosocosmicus*, ammonia-oxidizing archaea, comparative genomics, ammonium transporter, S-layer

## Abstract

Nitrification is a core process in the global nitrogen (N) cycle mediated by ammonia-oxidizing microorganisms, including ammonia-oxidizing archaea (AOA) as a key player. Although much is known about AOA abundance and diversity across environments, the genetic drivers of the ecophysiological adaptations of the AOA are often less clearly defined. This is especially true for AOA within the genus *Nitrosocosmicus*, which have several unique physiological traits (e.g., high substrate tolerance, low substrate affinity, and large cell size). To better understand what separates the physiology of *Nitrosocosmicus* AOA, we performed comparative genomics with genomes from 39 cultured AOA, including five *Nitrosocosmicus* AOA. The absence of a canonical high-affinity type ammonium transporter and typical S-layer structural genes was found to be conserved across all *Nitrosocosmicus* AOA. In agreement, cryo-electron tomography confirmed the absence of a visible outermost S-layer structure, which has been observed in other AOA. In contrast to other AOA, the cryo-electron tomography highlighted the possibility that *Nitrosocosmicus* AOA may possess a glycoprotein or glycolipid-based glycocalyx cell covering outer layer. Together, the genomic, physiological, and metabolic properties revealed in this study provide insight into niche adaptation mechanisms and the overall ecophysiology of members of the *Nitrosocosmicus* clade in various terrestrial ecosystems.

**IMPORTANCE:**

Nitrification is a vital process within the global biogeochemical nitrogen cycle but plays a significant role in the eutrophication of aquatic ecosystems and the production of the greenhouse gas nitrous oxide (N_2_O) from industrial agriculture ecosystems. While various types of ammonia-oxidizing microorganisms play a critical role in the N cycle, ammonia-oxidizing archaea (AOA) are often the most abundant nitrifiers in natural environments. Members of the genus *Nitrosocosmicus* are one of the prevalent AOA groups detected in undisturbed terrestrial ecosystems and have previously been reported to possess a range of physiological characteristics that set their physiology apart from other AOA species. This study provides significant progress in understanding these unique physiological traits and their genetic drivers. Our results highlight how physiological studies based on comparative genomics-driven hypotheses can contribute to understanding the unique niche of *Nitrosocosmicus* AOA

## INTRODUCTION

Since the discovery of the ammonia-oxidizing archaea (AOA), which belong to the archaeal phylum Thaumarchaeota, it has been clear that AOA are critical members involved in the global nitrogen cycle. This fact has been demonstrated by numerous ecological, physiological, and genomic studies of AOA over the past 20 years (1–5). In addition, AOA are often the most abundant ammonia-oxidizing microbes in natural environments, such as soil, freshwater, and marine environments (6–8). Phylogenetically, all AOA are classified as members of the class Nitrososphaeria and are placed in four order-level phylogenetic lineages, which correlate well with previously observed physiological traits: Nitrosopumilales (formally Group I.1a; abundant in marine and oligotrophic terrestrial environments), “*Candidatus* Nitrosotaleales” (Group I.1a-associated; abundant in acidic environments), Nitrososphaerales (Group I.1b; abundant in terrestrial environments), and “*Ca*. Nitrosocaldales” (thermophilic AOA clade; abundant in geothermal environments) (9). Recently, a fifth novel order-level lineage of AOA, the “*Ca*. Nitrosomirales” (abundant in marine and terrestrial environments), has been proposed (10). Presently, all cultured AOA grow as chemolithoautotrophs, using ammonia (NH_3_) as their sole or primary energy source and performing carbon fixation through the 3-hydroxypropionate/4-hydroxybutyrate pathway (1, 4).

AOA, along with ammonia-oxidizing bacteria (AOB) and complete ammonia-oxidizing bacteria (comammox), commonly co-exist, although their abundance ratios differ by environment (11–14). Such a diversity of ammonia oxidizers, all competing for the same substrate, ammonia (NH_3_) (15), in close proximity, raises many questions about the niche differentiation and physiological adaptations that allow for the coexistence of these microorganisms. Previous work has regarded differences in the whole-cell ammonia oxidation kinetics as a major driver of niche differentiation and competition among AOA, AOB, and comammox organisms. It was previously hypothesized that all AOA possess an extremely high affinity for NH_3_ as they frequently outnumber AOB in oligotrophic habitats (7, 16, 17). However, it has been recently observed that AOA possess a broad range of ammonia oxidation affinities and that these cellular kinetic parameters roughly align with the four phylogenetic orders of AOA. Interestingly, the Nitrososphaerales AOA (containing members of the *Nitrosocosmicus* genus) display substrate kinetics in the same range as many AOB (15, 18). With AOA possessing such a range of substrate affinities, it is no longer possible to strictly define the niche of AOA and AOB solely by these substrate kinetic parameters. Therefore, there must be other genomic and physiologic traits that contribute to defining the niche differentiation of AOA species.

Previously, comparative genomics has been successfully used to identify conserved or adaptative traits within the different lineages of AOA, which have provided insights into the inhabited ecological niches of the *Nitrosocaldus* AOA in hot spring environments (19, 20), the *Nitrosotalea* AOA in low pH environments (21), and the metabolic core genome as well as the metabolic flexibility of the *Nitrososphaera* AOA (22, 23). In addition, many culture-based studies have highlighted the differential responses of AOA to ecophysiological or biological factors (i.e., pH, light, temperature, metals, organic compounds, oxygen or substrate concentrations, reactive oxygen species, and other community members) (24–28). Although AOA within the genus *Nitrosocosmicus* are widespread and known to possess physiological features that set them apart from other AOA, a similar comparative genomic and physiological analysis is currently missing.

*Nitrosocosmicus* species are often described by traits that more closely resemble their AOB counterparts than other AOA species. For example, *Nitrosocosmicus* are comparatively large AOA cells, which display a high tolerance to high concentrations of both ammonium (NH_4_^+^) and nitrite (NO_2_^−^) (29). In addition to possessing the lowest affinity for NH_3_ among tested AOA species (15), it has also been noted that at least some members of the *Nitrosocosmicus* have a unique genomic repertoire for N_2_O production under low pH conditions (30) and may lack a canonical outermost surface layer (S-layer). Importantly, lacking an S-layer or possessing a unique S-layer among AOA may impact several of the previously mentioned observed cellular physiological traits as this one cellular property could directly or indirectly affect nutrient (NH_4_^+^, NO_2_^−^, NH_2_OH) capture, secretion, and/or transport (31, 32).

Therefore, in this study, we have conducted a comparative genomic analysis of the five publicly available closed *Nitrosocosmicus* genomes from cultured isolates in order to identify conserved genomic traits within this lineage of AOA. In addition, a more extensive genomic comparison with 39 AOA from all lineages was performed to gain deeper insights into the core metabolism of all AOA. Several physiologic experiments based on hypotheses generated by the comparative genomic study were also carried out with the isolated AOA strain *Nitrosocosmicus oleophilus* MY3. Together, this study highlights the power of functional genomics, combining *in silico* and culture-based approaches to identify and subsequently investigate the cellular ecophysiological adaptations of *Nitrosocosmicus* AOA.

## MATERIALS AND METHODS

### Cultivation of ammonia-oxidizing microorganisms

AOA (*N. oleophilus* MY3, *Nitrosotenuis chungbukensis* MY2, *Nitrosocosmicus franklandus*, and *Nitrososphaerea viennensis*) and the AOB (*Nitrosomonas europaea* ATCC19718) cultures were incubated in 100 mL of artificial freshwater medium (AFM) in 250 mL glass bottles (Duran, USA), under oxic conditions, in the dark, without shaking, at 30°C. The AFM components contained the following per liter: 0.2 g KH_2_PO_4_, 0.1 g CaCl_2_∙2H_2_O, 0.5 g KCl, 0.4 g MgCl_2_∙6H_2_O, and 1 g NaCl. After autoclaving, 1 mL nonchelated trace element solution (33), 1 mL NaFeEDTA solution (7.5 mM), 1 mL vitamin solution (15), 2 mM NaHCO_3_, and 10 mM HEPES were added per liter of AFM (15). The pH of the medium was adjusted to 7 with 1 M HCl and NaOH. Sodium pyruvate (0.5 mM) was added to all strain MY2 and *N. viennensis* cultures, and 1 mM NH_4_Cl was added to all cultures as the sole energy source unless stated otherwise. The concentrations of ammonium and nitrite were determined colorimetrically (15). For growth-based experiments, samples (1 mL) were taken at the indicated intervals for chemical or molecular analysis. All samples were stored at −80°C between sampling and analysis.

### Comparative genomic analysis

Genome sequencing and annotation of the five *Nitrosocosmicus* AOA have been described individually elsewhere and are available on GenBank with the following accession numbers: *N. oleophilus* MY3 (NZ_CP012850) (34), *N. franklandus* (GCA_900696045.1) (29), *Nitrosocosmicus hydrocola* G61 (CP017922) (35), *Nitrosocosmicus arcticus* Kfb (NZ_VOAH00000000.1) (36), and *Nitrosocosmicus agrestis* SS (VUYS00000000) (37). A broader comparative genomic analysis with an additional 34 complete or nearly complete AOA genomes, which have high completeness (>88%) but low contamination (<5%), was also performed. GenBank accession numbers and genome statistics are listed in Data File S1. For all 39 genomes analyzed, orthologous gene clusters were first identified to determine pan and core genomes of the *Nitrosocosmicus* and AOA, in general, using Get_Homologues with the default settings (38). BLASTP was used to pairwise align the core clusters using a 50% threshold for coverage and identity to identify the unique *Nitrosocosmicus* core genome.

Phylogenomic analysis of the 39 AOA was performed by following the IQ-TREE implemented within the Anvi'o phylogenomics workflow (39) with 71 concatenated ribosomal proteins of selected reference AOA strains obtained from the NCBI and Integrated Microbial Genomes and Microbiomes (IMG/M) databases. The constructed trees and operon arrangements were visualized using iTOL (v6.0) and used for annotation. Additionally, AOA genome similarity was also determined using genome-relatedness parameters, including average nucleotide identity (ANI) and average amino acid identity (AAI). ANI and AAI were performed using FastANI (40) and CompareM (https://github.com/dparks1134/CompareM), respectively.

### Measurement of hydroxylamine (NH_2_OH) concentration

NH_2_OH concentration was determined according to the method of Liu et al. (41). Briefly, 1.2 mL of a culture supernatant sample was supplemented with 75 µL of 160 mM sulfanilamide in 0.8 M HCl to remove any NO_2_^−^. The mixture was transferred to a 22 mL glass vial, and 4.8 mL deionized water was added, yielding a pH of 2. After that, 0.6 mL of 25 mM FeCl_3_ was added, and the vial was immediately closed gas-tight with a crimping tool. Control vials contained sample and water only to assess the background N_2_O present in the headspace and dissolved in the sample. The vials were shaken for 3 hours at 200 rpm and then transferred to an autosampler for N_2_O analysis on a gas chromatograph (GC) with an electron capture detector (ECD) as described in Liu et al. (41). NH_2_OH calibration in the range 0 to 1 µM was performed before each measurement.

### Expression of the ammonia transporter gene

For transcriptional analysis, strain MY3 was analyzed in three different conditions. Strain MY3 (1 L) was cultured in a 2 L glass bottle (Duran, USA) and supplemented with either a low (0.1 mM) or high (5 mM) concentration of NH_4_Cl. In addition, an ammonium starved condition was analyzed. Here, actively growing (~70% ammonia oxidized) cells from a 1 mM NH_4_Cl supplemented culture were harvested (4,000 × *g*, 10 min, 20°C) using 10 kDa cutoff Amicon Ultra-15 centrifugal filter unit (Merck Millipore, Germany), and the concentrated cells were washed with and resuspended in ammonia-free AFM for 24 hours before sampling. In all three conditions, single timepoint samples (500 mL of culture) were taken for RNA analysis. Cells were collected using a bottle top vacuum filtration system with a 0.1 µm mixed cellulose ester filter (ADVANTEC, Japan).

Total RNA was extracted using the PureLink RNA Mini Kit (Invitrogen, USA) according to the manufacturer's recommendations. Residual DNA was removed using the Recombinant DNase I (Takara, Japan). RNA was eluted in 30 µL of RNase-free water. cDNA was synthesized using the SuperScript IV First-Strand Synthesis System (Invitrogen, USA) with RNaseOUT solution (40 U µl^−1^; Invitrogen) according to the manufacturer's instructions. Concentrations of RNA and cDNA were determined using a DS-11 spectrophotometer (DeNovix, USA) in Bio-Health Materials Core-Facility, Jeju National University. qPCR samples were analyzed using the CFX Connect Real-Time PCR System (Bio-Rad, USA). Primer sets used for qPCR are listed in Table S1. The ammonia transporter gene expression level of strain MY3 was compared with the expression levels of the following genes: 16S rRNA gene, two enzymes involved in CO_2_ fixation (methylmalonyl-CoA mutase large subunit and 4-hydroxybutyryl-CoA dehydratase), the ammonia monooxygenase subunit A (*amoA*) gene, and the RNA polymerase subunit B (*rpoB*). Absence of genomic DNA in RNA samples was confirmed by the lack of qPCR amplification of the RNA samples with the same primers.

### Cryo-electron tomography

The actively growing (1 L) strain MY3, *N. franklandus*, and *N. viennensis* from late-exponential phase was concentrated to 1 mL using a vacuum filtration system with a 0.1 µm polyethersulfone (PES) membrane filter (GVS, USA). The cell suspension (2.5 µL) was applied to a quantifoil R2/2 Cu holey carbon grid (Quantfoil Micro Tools), which was previously glow-discharged. The EM grid was plunge-frozen into liquid ethane using a Vitrobot Mark IV (Thermo Fisher Scientific) with settings of 95% humidity, 4°C, and 5 second blotting time. Images of the ice-embedded cells were obtained using a Titan Krios G4 microscope (Thermo Fisher Scientific) and a Falcon 4i direct electron detector (Thermo Fisher Scientific) at the Core Research Facilities of Pusan National University. The images were recorded at a nominal magnification of 18,000×, resulting in an imaging resolution of 4.429 Å per pixel. For electron tomography, tilt series images were collected at ±50° in 3° increments. The total electron dose for the tilt series was 140 e−/Å^2^ using a low-dose mode. The tilt series were aligned using gold fiducials, and tomograms were reconstructed using SIRT in the IMOD software (42).

## Results and Discussion

### Phylogenomic placement of the *Nitrosocosmicus* lineage

**Fig 1:**
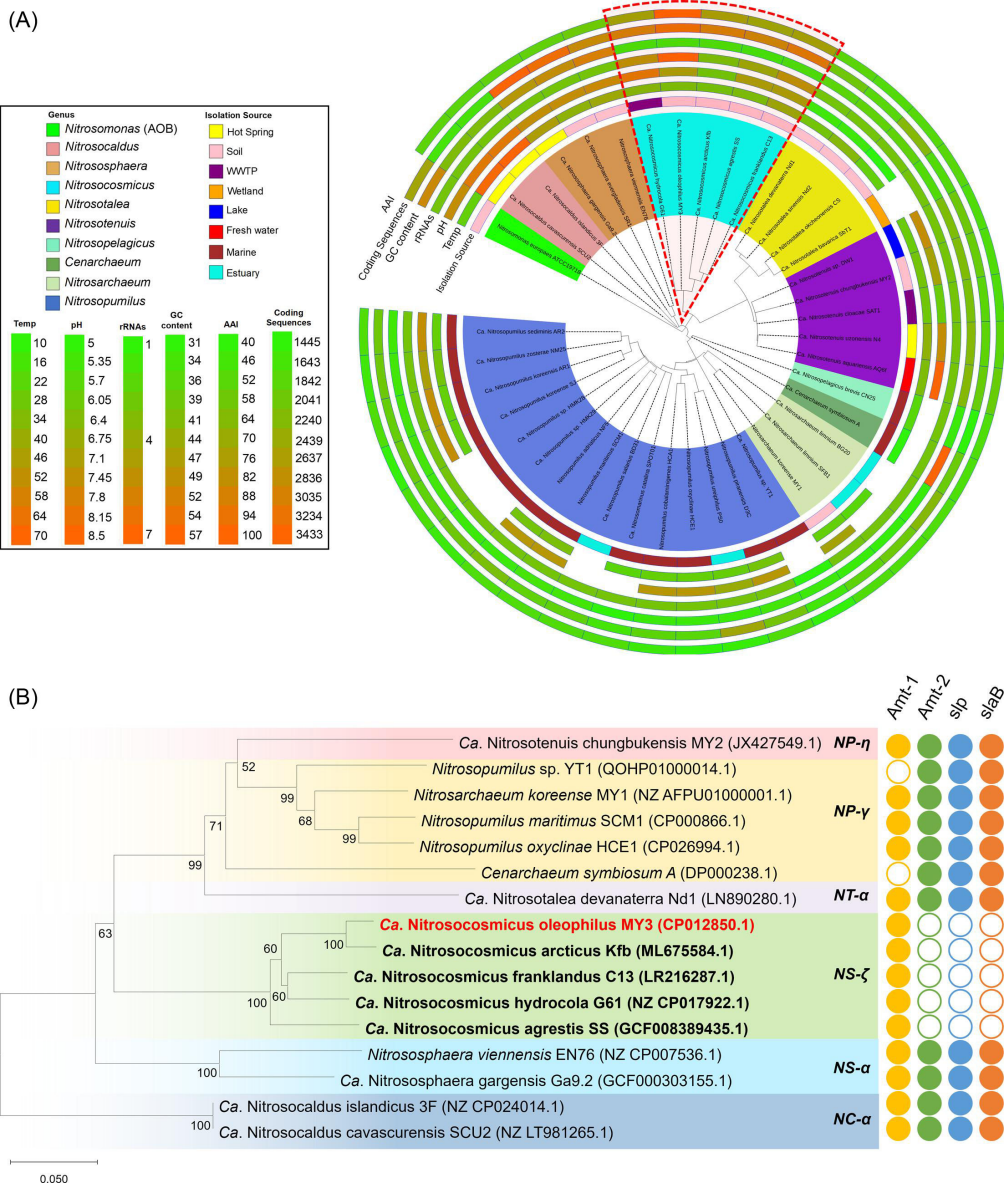
(**A**) Phylogenetic clustering of 39 AOA species based on the concatenation of 71 core genes. Tree leaves are colored by genera belonging to the order Nitrosopumilales (*Nitrosopelagicus*, *Creanarchaeum*, *Nitrosarchaeum*, and *Nitrosopumilus*), Nitrosotaleales (*Nitrosotalea*), Nitrososphaerales (*Nitrososphaera* and *Nitrosocosmicus*), and Nitrosocaldales (*Nitrosocaldus*). Genotypic and phenotypic parameters, including coding sequences, amino acid identity (AAI), G+C percentage, number of rRNAs (5, 16, and 23S), optimal pH, and optimal temperature, are shown in the red and green matrices (from outside in). Distinct colors in the innermost ring represent isolation sources of individual AOA species. Blank spaces in the circles represent the unavailability of data. The tree is rooted with the AOB *Nitrosomonas europaea* ATCC19718, and tree branches represent phylogenetic distances. (**B**) Comparative phylogenetic analysis of archaeal *amoA* gene sequences (~570 bp) from described AOA representatives constructed using the Neighbor-Joining method, and confidence values are based on 1,000 bootstrap replications. The presence or absence of ammonium transporters (Amt-1 and Amt-2), S-layer protein (*slp*), and cell-anchoring gene (*slaB*) in the AOA genomes is indicated with solid and hollow circles, respectively. The *amoA* gene clade names are derived from Alves et al. (5).

Phylogenetic clustering of 39 AOA genomes was performed based on the concatenation of 71 core orthologous genes. Only AOA genomes with an isolated or enriched laboratory culture were selected for phylogenetic analysis and comparative genomic analysis in this study. The phylogenetic placement of the 39 AOA genomes into each of the four groups of AOA was consistent with previous studies (5), and all five *Nitrosocosmicus* genomes considered in this study clustered closely together and within the genus *Nitrososphaera* (Fig. 1A). Originally, *Nitrosocosmicus* AOA were grouped with members of the genus *Nitrososphaera* as a “*Nitrososphaera* sister cluster” (43) but have been recognized more recently as a distinct genus based on physiological properties (15, 29, 34) and phylogenetic genome evolution (44). A similar phylogeny was obtained from the ammonia monooxygenase subunit A (*amoA*) gene, which placed all *Nitrosocosmicus* members in the archaeal *amoA* gene clade NS-ζ (5) (Fig. 1B). The five *Nitrosocosmicus* AOA displayed >90.1% and 99.5% similarity based on *amoA* gene and AmoA protein sequence analysis, respectively. Furthermore, all five shared >72% average nucleic acid identity (ANI) and >68% average amino acid identity (AAI) at the genome level. The ANI and AAI to all other AOA were below 66% and 58%, respectively, indicating a genus-level distinction for *Nitrosocosmicus* (Fig. S1).

### General genomic traits of the *Nitrosocosmicus*

The five *Nitrosocosmicus* AOA genomes all share distinct traits that distinguish them from other AOA species, including having comparatively large genomes (ca. 3.0 Mb of average size). While similar in size to other *Nitrososphaera* (ca. 2.9 Mb), genomes of *Nitrosocosmicus* AOA are almost double that of the *Nitrosopumilales* (ca. 1.7 Mb), *Nitrosotaleales* (ca. 1.5 Mb), and *Nitrosocaldales* (ca. 1.6 Mb) AOA analyzed in our data set. This increased genome size has previously been attributed to a substantial increase in coding sequences linked to adaptive survival strategies for terrestrial environments (44). In addition to sheer genome size, *Nitrosocosmicus* members are also the only cultured AOA to date to possess multiple rRNA gene copies/operons (Fig. 1A ; Data File S1). Higher rRNA gene copy numbers may indicate a need for more ribosomes at any given time in these *Nitrosocosmicus* species or a need to be able to increase ribosome production/concentration at a faster rate than other AOA (45, 46). While the *in situ* physiological advantage of this is currently unknown, it can be speculated that generating active ribosomes faster would be advantageous in environments where growth conditions are highly variable (47, 48). In addition, it has been speculated that encoding unlinked or non-operon rRNA gene copies may eliminate the need for RNaseIII processing and, therefore, limit phage infections that are dependent on using cellular RNaseIII (48). However, little is known about the transcriptional patterns of these multiple rRNA gene copies and what physiologic, defensive, or metabolic adaptive traits they may confer within the *Nitrosocosmicus*. Although mutation studies in these species are not yet (widely) available, the transcription and function of these additional rRNA copies warrant further investigation.

**Fig 2:**
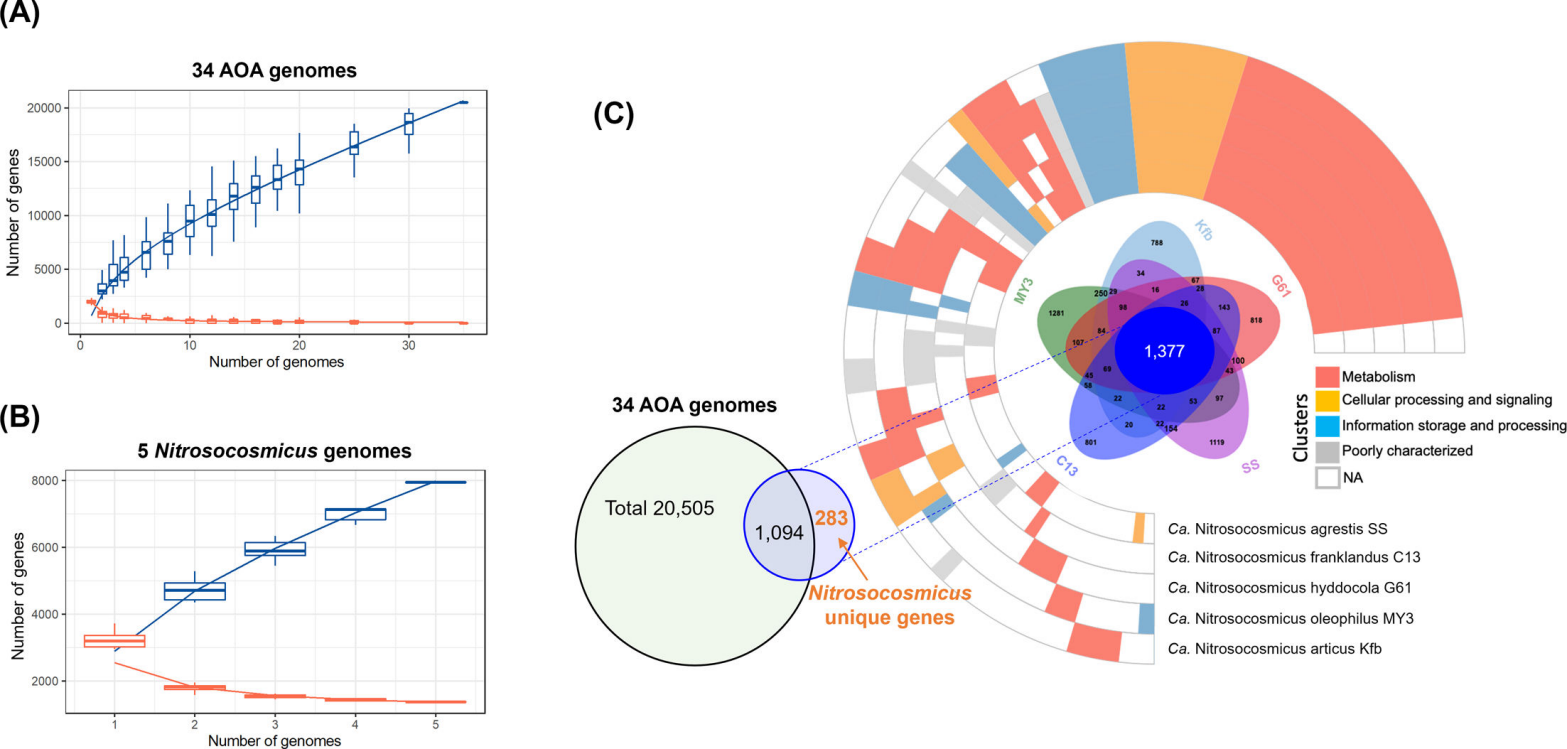
Comparative pan (blue) and core (orange) genome analysis of 34 AOA genomes (**A**) and five *Nitrosocosmicus* genomes (**B**)**.** (**C**) The core *Nitrosocosmicus* genome is represented by a Venn diagram encircled by the functional representation of all the gene clusters. In addition, the *Nitrosocosmicus* core genome that is also found within 34 AOA genome clusters, and the unique core genes are shown.

To provide further insights into the conserved genetic repertoire within the *Nitrosocosmicus* AOA, we determined the core genome and the unique core genome of the *Nitrosocosmicus*. Here, the core genome is composed of genes shared by all five *Nitrosocosmicus* AOA strains. The unique core genome is a subset of the core genome, indicating that the genes were absent from all other non-*Nitrosocosmicus* 34 AOA genomes analyzed. In order to make these comparisons, we determined the core and pangenomes of the five *Nitrosocosmicus* AOA and 34 other AOA (Fig. 2). In the pangenome of the 34 non-*Nitrosocosmicus* AOA, there were 20,505 total genes, with just 237 core genes (Data File S1). The core genes included the genes assumed to be responsible for ammonia oxidation (36) and for carbon fixation through the 3-hydroxypropionate/4-hydroxybutyrate pathway (49). Interestingly, of the 237 core genes, ~28% are hypothetical or have no predicted function, highlighting how much is still unknown about even the most conserved genomic features of the AOA. As there are only five *Nitrosocosmicus* included in the current study, the core genome of the *Nitrosocosmicus* is comparatively larger, containing 1,377 core genes out of a total pangenome of 7,947 genes (Fig. 2; Data File S1).

### The *Nitrosocosmicus* core and unique core genome

As all five *Nitrosocosmicus* species are closely related, a large portion of the core genome comprises genes responsible for general cellular function, such as rRNA subunits, NADH oxidoreductase subunits, and transcription factors/regulators. Also, as is the case for the core genome of the other 34 AOA analyzed, the five *Nitrosocosmicus* also contained the expected genes responsible for ammonia oxidation (*amoA*, *amoB*, *amoX*, *amoY*, and *amoZ* but not *amoC*) and carbon fixation through the 3-hydroxypropionate/4-hydroxybutyrate pathway. In addition, the *Nitrosocosmicus* core genome contains several genes potentially involved in energy conservation or potentially electron transfer (including genes encoding multicopper oxidases and blue copper domain-containing proteins) along with the complete set of urease genes (*ureA, ureB,* and *ureC*), the urease accessory proteins (*ureE, ureF, ureG,* and *ureD*), and a urea transporter (Data File S1). Notably, all subunits of V-type ATPase were also present in the core genome, indicating that all the *Nitrosocosmicus* AOA have a survival advantage in acidic conditions like previously characterized acidophilic AOA (50).

In addition to the core genome of the five *Nitrosocosmicus* AOA (1,377 genes), these *Nitrosocosmicus* species share a unique core genome of 283 genes, which are not found in any of the other 34 AOA investigated (Fig. 2; Data File S1). The unique core genes fall within a wide variety of COG functions, and ~43.5% of the COGs were assigned to specific functional categories with amino acid transport and metabolism (E, ~4.9%), coenzyme transport and metabolism (H, ~4.6%), general function (R, ~4.2%), translation, ribosomal structure and biogenesis (J, ~3.9%), and energy production and conversion (C, ~3.5%), being among the most abundant (Table S2). In addition, *nosD,* which is a periplasmic protein required for the maturation of copper-containing nitrous oxide reductases (51), is also in the *Nitrosocosmicus* unique genome. Its presence warrants the need to verify if *nosD* indeed functions in the maturation of a candidate copper-containing nitrous oxide reductase in *Nitrosocosmicus* AOA in future studies, as previous studies have shown nitrous oxide reductase activity under low pH conditions in *Nitrosocosmicus* strain MY3 (15). The *Nitrosocosmicus* unique genome also contains multiple copies of glycosyltransferase genes, which may be related to the makeup of the outermost surface structure of *Nitrosocosmicus* species (see the surface structure below for details) (22, 34). Furthermore, the exopolysaccharide synthesis genes related to cell aggregation and biofilm formation in strain MY3 (34) enable cells to resist physical stresses (52) and attach to surfaces, allowing cells to avoid being thoroughly washed away in wastewater treatment plants (WWTPs). However, unlike AOA strains in another cluster (NS-*α*) of Nitrososphaerales, gene clusters responsible for the assembly of flagella and chemotaxis were not identified in any of the *Nitrosocosmicus* genomes.

It is important to mention that only five *Nitrosocosmicus* genomes have been analyzed in this comparison, and the unique/conserved genome repertoire will only shrink as more *Nitrosocosmicus* genomes from cultured representatives become available. However, several genes were already noticeably absent from the *Nitrosocosmicus* core genome. First, while all five *Nitrosocosmicus* genomes encoded a low-affinity type ammonium transporter (Amt-1), no high-affinity type ammonium transporter (Amt-2) was encoded (Fig. 1B; Data File S1). In addition, no *Nitrosocosmicus* species analyzed encoded detectable homologs for the S-layer proteins or cell anchoring proteins (which connect the S-layer to the inner membrane) found in most other AOA (Fig. 1B) (31). The physiological manifestations of these two genomic characteristics were investigated with culture-based experiments and are discussed in great detail below in the ammonium transporter gene expression and surface structure sections, respectively.

### Ammonium transporter (*amt*) gene expression

All complete AOA genomes investigated encode at least one ammonium transporter, with most AOA encoding both a low-affinity type (Amt-1) and a high-affinity type (Amt-2) transporter (Fig. 1B) (53). With all AOA being dependent on ammonia for energy generation and ammonia/ammonium being in constant flux in many environments, the possession of multiple transporters that can be independently regulated is likely a beneficial physiologic adaptation. In previous studies, low ammonia/ammonium concentrations led to high expression of the high-affinity and depression of expression of the low-affinity type ammonium transporters in *Nitrosopumilus maritimus* (54, 55). This regulation highlights how *N. maritimus* is able to maximize substrate capture. However, it is unclear whether ammonia/ammonium transport in AOA and other ammonia oxidizers is generally related to energy-generating processes (e.g., by storing substrate intracellularly) or only required for cellular nitrogen assimilation.

**Fig 3:**
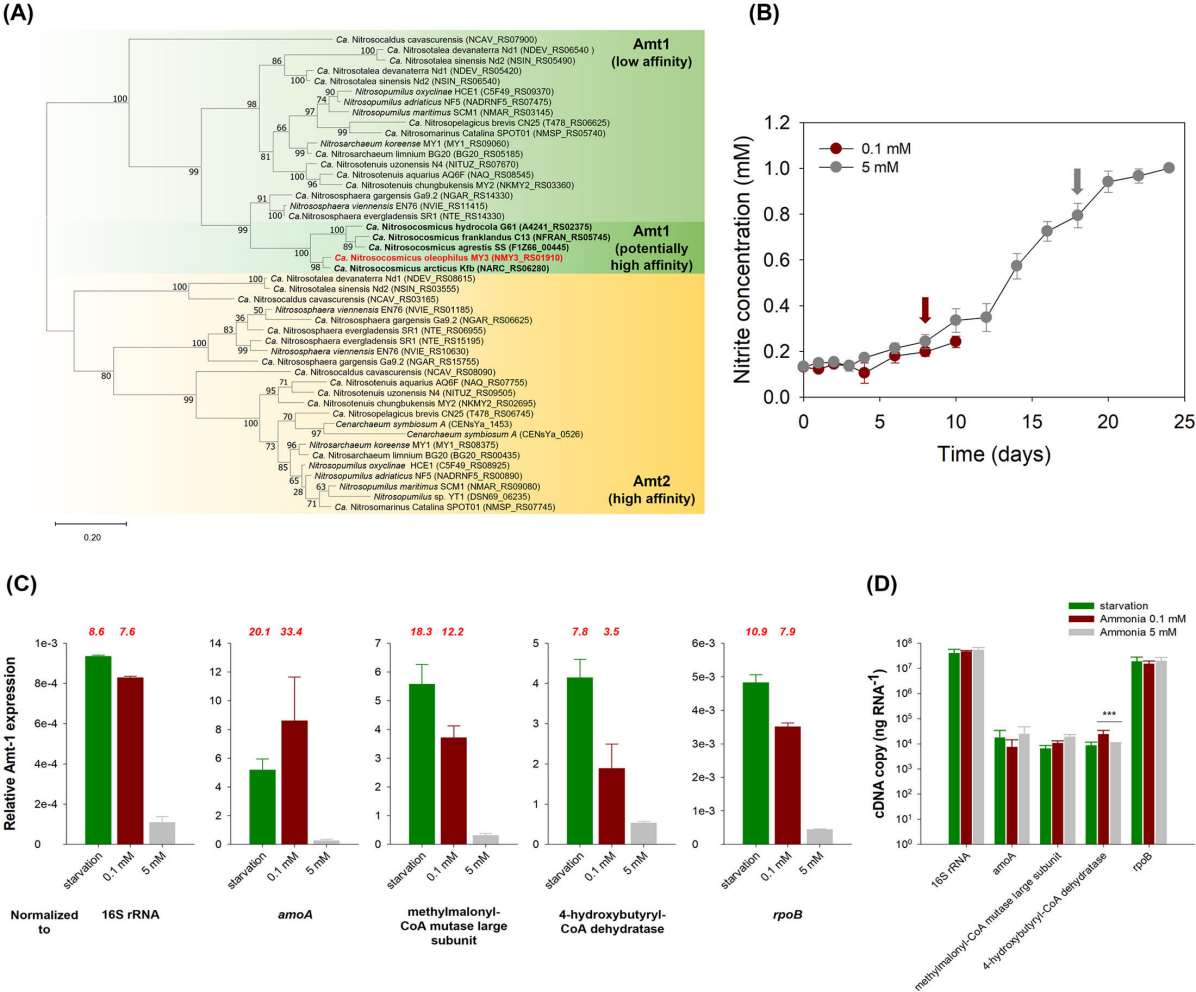
The ammonium transporter (Amt) of *Nitrosocosmicus oleophilus* MY3 is upregulated under low ammonium conditions. (**A**) A comparative phylogenetic analysis of Amt amino acid sequences from AOA genomes. The tree was inferred by the maximum-likelihood method using an LG + F model and an ultrafast bootstrap value of 1,000. (**B**) Cultures were initially supplemented with 0.1 or 5 mM ammonium chloride, and samples for RNA were taken during log phase activity for the 0.1 mM (red arrow) and 5 mM (gray arrow) ammonia cultures. The initial cell density after inoculation was ~1.5 × 10^5^ cells ml^−1^. Values represent the average and standard deviation nitrite concentration of triplicate cultures. (**C**) The *amt* gene expression ratios normalized to four genes across at all tested initial ammonium concentrations. The values represent the average and standard deviation of duplicate qPCR experiments from triplicate cultures. The relative expression fold ratio compared with the 5 mM ammonia condition is indicated above the graphs in red and is significantly higher in all cases (*P* < 0.05). (**D**) The transcript abundance of 16S rRNA, *amoA*, two CO_2_ fixation genes (methylmalonyl-CoA mutase large subunit and 4-hydroxybutyryl-CoA dehydratase), and *rpoB* were normalized to the 16S rRNA gene copy number of the cultures used for cDNA preparation. The values represent the averages and standard deviation of duplicate qPCR experiments from triplicate cultures. The significant difference between low and high ammonia conditions for each gene was determined by a *t*-test (***: *P* < 0.05).

In contrast to most other AOA, all fully sequenced *Nitrosocosmicus* AOA, including strain MY3, possess only the low-affinity type transporter (Fig. 1B and 3A; Fig. S3). Therefore, it could be hypothesized that *Nitrosocosmicus* AOA would be less efficient in acquiring extracellular ammonium than AOA (i.e., *N. maritimus*) with a high-affinity type transporter or that low ammonium concentrations would not trigger the transcription of the low-affinity transporter. Indeed, low-affinity ammonium transport might already be reflected in the observed physiology of several *Nitrosocosmicus* AOA strains, which often display very high ammonium tolerance (29, 34, 35, 37) and low ammonia affinities (15) compared with other AOA.

The expression of Amt-1 (MY3_00435) in strain MY3, in response to a period of starvation, low, and high initial ammonium concentrations (0, 0.1, and 5 mM), was investigated in this study (Fig. 3). The low-affinity Amt-1 was expressed under all three tested conditions and its relative expression was normalized to that of four genes, whose expression was stable on a per nanogram RNA basis for all three treatments (Fig. 3D). Amt-1 expression was highest under the starvation or 0 mM ammonium condition and decreased with increasing initial ammonium concentrations (Fig. 3C). Compared to the high (5 mM ammonium) substrate treatment, Amt-1 expression was 3.5 to 33.4 times higher in the low (0.1 mM ammonium) substrate treatment and was 7.8 to 20.1 times higher in the starvation treatment (Fig. 3C). In agreement with this finding, the same Amt-1 expression trend was previously observed with *Nitrosocosmicus agrestis*, where upregulation of a low-affinity Amt-1 transporter occurred under lower ammonium conditions (7.96 µM) compared with high ammonium conditions (796 µM) (37).

Although the overall ammonium transporter expression profile among AOA is similar, low substrate conditions result in high ammonium transporter expression. The Amt-1 expression profile in *Nitrosocosmicus* species is in stark contrast to the Amt-1 expression profile previously observed in marine AOA (54, 55). This may suggest that the Amt-1 of *Nitrosocosmicus* strain MY3 has a higher affinity (lower *K*_*m*_) than expected, functioning more like a high-affinity Amt-2 transporter. However, in order to confirm this, the kinetic properties of Amt-1 transporters from a collection of AOA species would need to be investigated. The question of ammonium transporter affinity posed here adds to the many current open questions regarding whether ammonia or ammonium is transported, if/how H^+^ are cotranslocated, and what the Amt transporters are responsive to (assimilation vs. energy generation needs) (56, 57). It is also possible that, as the sole ammonium transporter in *Nitrosocosmicus*, the expression profile of Amt-1 highlights the lack of flexibility available compared to AOA, which encode multiple ammonium transporters. This would imply that even as a low-affinity transporter, Amt-1 is still able to participate in ammonium scavenging at low concentrations in the *Nitrosocosmicus*.

### The surface structure of *Nitrosocosmicus* strain MY3

The S-layer is a proteinaceous macromolecular aggregate that forms a crystal lattice on the cell surface of most archaea and some bacteria (58, 59) and serves as the first selective barrier between the external environment and the cell interior (60). The S-layer also enables cell attachment to solid surfaces and is considered a critical factor in biofilm formation (61). Most archaea possess several identifiable S-layer genes in their genome and an imageable S-layer outermost structure. This includes several AOA from both marine (*N. maritimus*) and soil environments (*N. viennensis*, *Nitrosotalea devanaterra*) (62–64). Here, the electrochemical properties of the S-layer in AOA were even proposed to aid in the capture and concentration of ammonium from the environment (31, 32). However, all *Nitrosocosmicus* AOA to date, including strain MY3, lack any identifiable canonical S-layer proteins (*slp*) or cell-anchoring gene (*slaB*) that are found in all the other groups of AOA from varying environments (Fig. 1B) (65).

**Fig 4:**
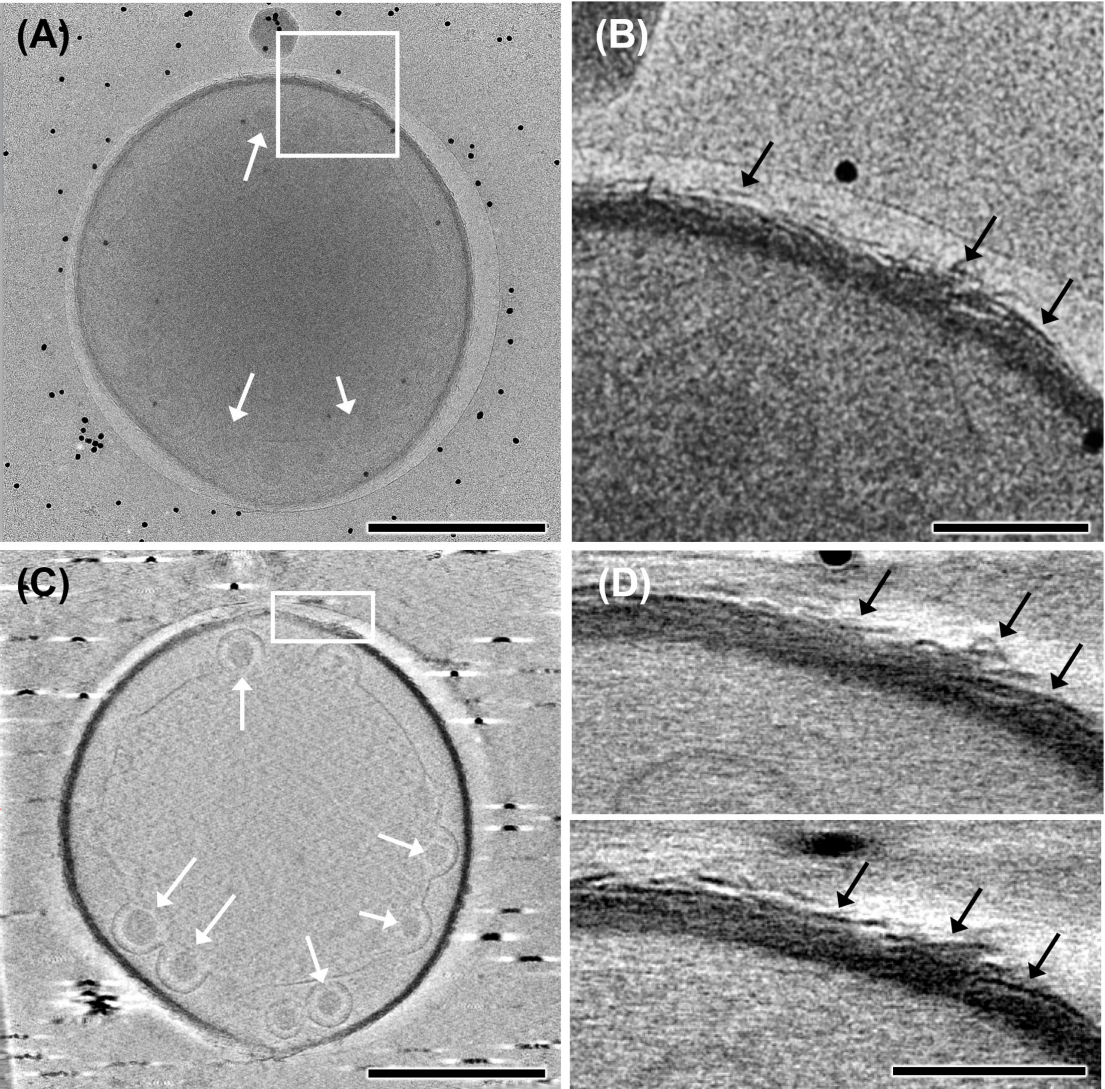
Cryo-electron tomography analysis of *N. oleophilus* MY3 cell. (**A**) A whole-cell image of strain MY3 (Video S1). (**B**) An enlargement of the rectangular in panel A. (**C**) A representative XY slice from the electron tomographic slices (Video S2). (**D**) Two different XY slices of the rectangle region in panel C. The black arrows in panels B and D indicate the hair-like structures on the cell surface, and the white arrows in panels A and C indicate the vesicle-like structures in (pseudo)periplasmic space. Scale bar equals 500 nm in panels A and B and 100 nm in panels C and D.

Therefore, cryo-electron tomography was used to investigate whether *Nitrosocosmicus* AOA species are truly missing a visible outer S-layer structure and/or if another form of external cell structure, coat, or matrix could be identified. For comparison, the outer structures of *N. franklandus* and *N. viennensis* were also analyzed. *N. viennensis* encodes a canonical S-layer protein and cell-anchoring genes and has a previously characterized visible S-layer (64). Imaging of *N. viennensis* clearly shows a crystal lattice-like outer S-layer in agreement with previous observations (Fig. S4) (64). In stark contrast, *Nitrosocosmicus* MY3 and *N. franklandus* both appear to have very thick cell walls (~18 nm), but no structure resembling a lattice-like S-layer was observed on the surface of either species (Fig. 4; Fig. S4). However, thin filaments or hair-like structures (~30 nm) were observed covering the cell surface of both *Nitrosocosmicus* species and are visually similar to glycoprotein and/or glycolipid-based glycocalyx cell coverings that have been previously described in the microalgae *Coelastrella* (66, 67). Similar to the hyperthermophilic archaeon *Ignicoccus*, with the absence of an S-layer (68, 69), an inner membrane structure is visible as a layer with two distinguishable electron-dense layers for each membrane in both strain MY3 and *N. franklandus* (Fig. 4; Fig. S5). In addition, vesicle-like structures are visible in the cytoplasm, which have also been observed in *Ignicoccus* (69, 70). The functional significance of these vesicles in *Nitrosocosmicus* is currently unknown. However, these vesicles could function in a manner similar to vesicle-mediated processes in eukaryotic cells, aiding in the cellular functions associated with the double-membrane structure of *Nitrosocosmicus* (71, 72). They could contribute in the import and/or export processes that may be involved in the transport of genomic DNA for genetic material exchange or proteins destined for the outer membrane (69). Therefore, further studies into the role of inner vesicle-like structures within the *Nitrosocosmicus* are warranted.

In agreement with the observation that *Nitrosocosmicus* species may have a glycocalyx cell covering, the genomes of all five *Nitrosocosmicus* species (including the two visualized here) encode multiple glycosyltransferase genes and several amino sugar transformation genes (in Data File S1). Taken together, it is possible that *Nitrosocosmicus* species have an outermost layer that resembles a glycocalyx cell covering coated with amino sugars. While no conclusive evidence on the outer layer structure of *Nitrosocosmicus* species was determined, the presence of a canonical S-layer was not identified in multiple *Nitrosocosmicus* species. An amino sugar-enriched glycocalyx outermost layer would likely play various physiological roles, including acting as a molecular sieve and aiding in cell adhesion and possibly biofilm formation under diverse environmental conditions, such as elevated ammonia concentrations (see Supplemental Information; Fig. S6) (73–77). The exact components of what the outermost layer of *Nitrosocosmicus* species is composed of are of particular interest, as the energy-generating pathway in AOA is presumed to occur on the periplasmic side of the cytoplasmic membrane or in the (pseudo)periplasm itself. Therefore, the composition of the outermost surface may affect the ability of *Nitrosocosmicus* species to trap or retain ammonium (15, 65) or other essential reactive intermediates in the ammonium oxidation pathway such as hydroxylamine (78).

### Analysis of net NH_2_OH concentration during ammonia oxidation

In all three currently recognized groups of ammonia oxidizers (AOA, AOB, and comammox), the first step of ammonia oxidation is carried out by an ammonia monooxygenase (AMO) enzyme, generating hydroxylamine (NH_2_OH) as an essential intermediate (41, 79, 80). However, the mechanism(s) and enzymatic machinery responsible for the oxidation of NH_2_OH to NO_2_^−^ remain unclear. This is especially true within the AOA, where no identifiable ortholog of the bacterial hydroxylamine dehydrogenase (HAO), responsible for the conversion of NH_2_OH to NO, has been identified (81, 82). Therefore, the process of NO_2_^−^ production from NH_2_OH in AOA is highly speculative and has been proposed to occur through two different mechanisms: (i) NH_2_OH is oxidized via NO to NO_2_^−^ exactly as proposed in the AOB with currently uncharacterized enzymes (83) or (ii) NH_2_OH and NO are converted to two NO_2_^−^ molecules by an uncharacterized copper-based enzyme complex (78). In both cases, hydroxylamine oxidation is expected to occur in the (pseudo)periplasmic space. Therefore, differences in outer cell surface coatings may affect the amount of NH_2_OH loss or leakage from different AOA lineages.

**Fig 5:**
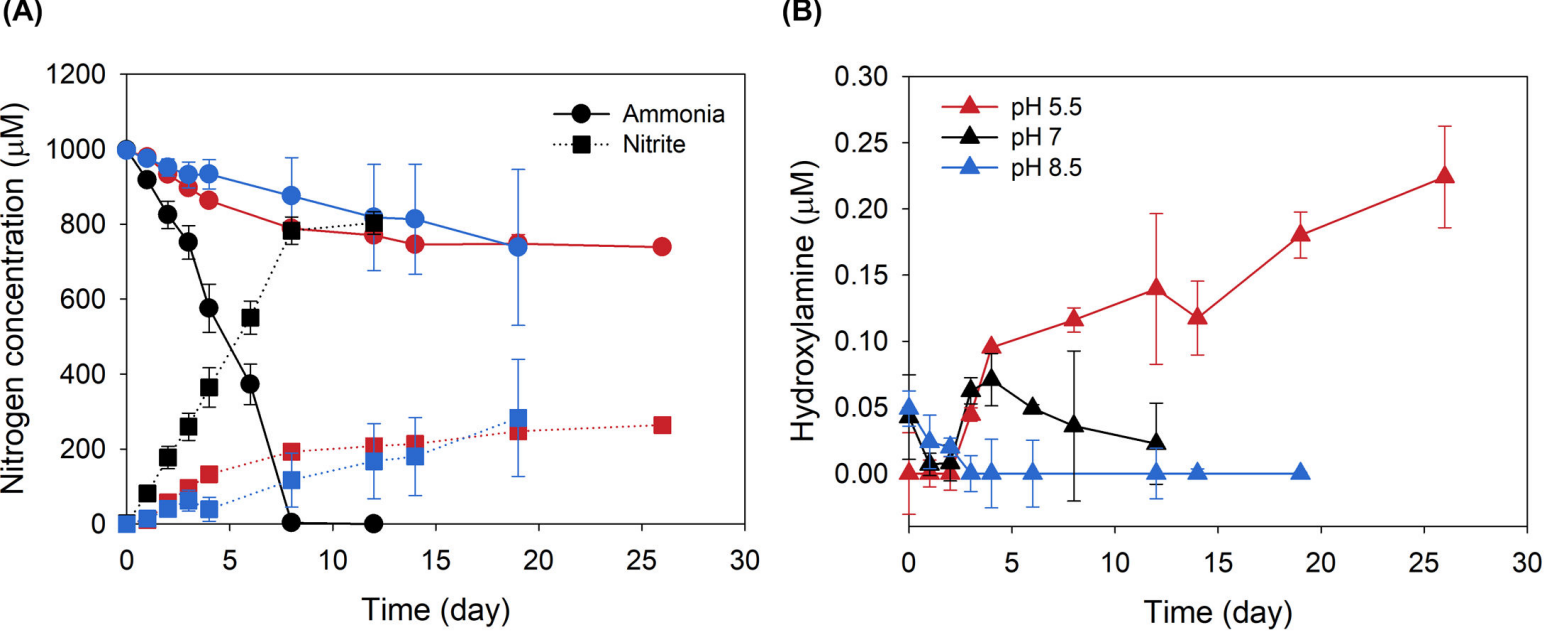
Hydroxylamine accumulation in *Nitrosocosmicus* MY3 during ammonia oxidation. The influence of initial culture medium pH on ammonia oxidation and nitrite production (**A**)**,** along with net hydroxylamine accumulation (**B**)**,** was determined. The values represent the average and standard deviation of triplicate cultures.

To investigate if AOA from the *Nitrosocosmicus* genus have a distinct NH_2_OH loss profile, we measured net NH_2_OH concentrations during ammonia oxidation by strain MY3 at pH 5.5, 7, and 8.5. At the neutral pH condition (pH 7), the optimum growth pH for strain MY3, NH_2_OH concentrations initially decreased, increasing during the exponential phase of ammonia oxidation up to 0.07 µM, and finally decreasing again as ammonium was completely oxidized (Fig. 5). While the overall trend of measurable extracellular NH_2_OH was different, the net extracellular NH_2_OH measurable concentrations were not higher than what has been reported for other AOA species (>0.25 µM) and is far less than have been reported for AOB (>1 µM) or comammox (~0.4 µM) (41). Although culture activity (speed of ammonia oxidation) and environmental factors likely factor into net NH_2_OH loss, it does not appear that the lack of an S-layer (Fig. 4) leads to a dramatic increase in NH_2_OH loss during ammonia oxidation from AOA within the genus *Nitrosocosmicus* (Fig. 5).

Perhaps unsurprisingly, the ammonia oxidation activity and net NH_2_OH profile of strain MY3 differed when the initial cultivation medium pH was modified. In both the low and high pH conditions, only about 200 µM ammonium was oxidized over the course of the experiment. At a high initial pH (pH 8.5), ammonia oxidation proceeded slowly, and no measurable NH_2_OH accumulation was detected. In contrast, at a low initial pH (pH 5.5), the extracellular NH_2_OH concentration continued to increase to ~0.25 µM over the course of the 26-day experiment, even when the rate of total ammonium oxidation had almost stopped entirely (Fig. 5). This may highlight the difference between slow total ammonia oxidation activity (pH 8.5) and the inhibition of hydroxylamine oxidation before ammonia oxidation at low pH (pH 5.5). This could be attributed to the following reasons: (i) NH_2_OH is an unstable and highly reactive compound due to the presence of a labile O-N bond; therefore, the produced NH_2_OH is rapidly converted to nitric oxide (NO) and then NO_2_^−^ or other nitrogen-containing compounds such as N_2_O at pH 8.5 (82, 84) due to its p*K_a_* value of 6.03. In contrast, at pH 5.5, NH_2_OH exists mostly in its protonated form, NH_3_OH^+^, which is much more stable and slowly decomposed to other nitrogen compounds. (ii) The optimum pH of NH_2_OH-oxidizing or converting enzymes would be much higher than pH 5.5, therefore at pH 5.5, resulting in poor enzyme activity, although the enzyme responsible for the NH_2_OH conversion in AOA has not been clearly identified yet (85).

In addition to being a central and essential intermediate during ammonia oxidation, NH_2_OH is also a crucial precursor for N_2_O production by AOA. According to previous studies with strain MY3, the enzymatic reduction of NO_2_^−^ through a cytochrome P450 pathway was enhanced at a lower pH, where NH_2_OH has increased stability (30). Here, this enhanced enzymatic NO_2_^−^ reduction process was independently reconfirmed with AOA strains MY2 and MY3 using an ^18^O tracing method (Fig. S2; see details in Supplemental Information).

### Conclusions

As a group, AOA within the genus *Nitrosocosmicus* have been previously observed to display physiological traits that separate them from other AOA (i.e., high tolerance to extracellular ammonium/nitrite, the lowest measured substrate affinities, and large cell size). While seemingly distinct, all these traits could be influenced by diffusion through, attraction to, and transport over the outer and inner cell membranes. Our study provides two examples of the physiological uniqueness of *Nitrosocosmicus* AOA cells. First, genomic and morphological evidence indicates that *Nitrosocosmicus* AOA do not encode or possess a crystal lattice-like outermost layer (S-layer), unlike other AOA. This absence very likely impacts nutrient capture, secretion, and transport dynamics, which could be essential for their adaptation to various environmental conditions. In addition, this one trait may provide an explanation for several of their physiological features that are unique among the AOA. Secondly, the transcriptional expression profiles of the low-affinity ammonium transporters in *Nitrosocosmicus* AOA contrast with those of other AOA, suggesting a unique mechanism for managing ammonium uptake under different environmental conditions. By leveraging their unique physiological traits, we can better utilize archaeal nitrification processes to enhance environmental management practices. Our results highlight how physiological studies based on comparative genomics-driven hypotheses can contribute to understanding the unique niche of *Nitrosocosmicus* AOA.

## SUPPLEMENTAL MATERIAL

Data File S1 mbio.02169-24-s0001.xlsx General information for Fig. 1, core and unique genes, key genes, and glycosyltransferase.

Supplemental information mbio.02169-24-s0002.docx Supplemental text, figures, tables, and video legends.

Video S1 mbio.02169-24-s0003.mp4 3D of *Nitrosocosmicus oleophilus.*

Video S2 mbio.02169-24-s0004.mp4 3D of *Nitrososphaera viennensis.*

Video S3 mbio.02169-24-s0005.mp4 3D of *Nitrosocosmicus franklandus.*

ASM does not own the copyrights to Supplemental Material that may be linked to, or accessed through, an article. The authors have granted ASM a non-exclusive, world-wide license to publish the Supplemental Material files. Please contact the corresponding author directly for reuse.
