# Supplementary material for: Adaptive traits of Nitrosocosmicus clade ammonia-oxidizing archaea
Source: mBio. 2024 Oct 3;15(11):e02169-24. doi: 10.1128/mbio.02169-24 (PMC11559005; doi:10.1128/mbio.02169-24)
Supplement: Supplemental information — Supplemental text, figures, tables, and video legends. [file mbio.02169-24-s0002.docx]

**Supplementary information**

**Adaptive traits of *Nitrosocosmicus* clade ammonia-oxidizing archaea**

Saem Han^1#^, Seongwook Kim^1#^, Christopher J. Sedlacek^2*^, Adeel Farooq^3^, Chihong Song^4^, Sujin Lee^4^, Shurong Liu^5^, Nicolas Brüggemann^6^, Lena Rohe^7^, Miye Kwon^8^, Sung-Keun Rhee^9^, and Man-Young Jung^1, 3*^

^1^Interdisciplinary Graduate Program in Advance Convergence Technology and Science, Jeju National University, 102 Jejudaehak-ro, Jeju 63243, South Korea

^2^Department of Biology Education, Jeju National University, 102 Jejudaehak-ro, Jeju 63243, Republic of Korea

^3^Division of Microbial Ecology, Centre for Microbiology and Environmental System Science, University of Vienna

^4^Core Research Facility, Pusan National University, Yangsan 50612, Republic of Korea

^5^School of Agriculture, Sun Yat-Sen University, Shenzhen 518107, China

^6^Agrosphäre (IBG-3), Institut für Bio- und Geowissenschaften (IBG), Forschungszentrum Jülich GmbH, Jülich 52428, Germany

^7^Thünen Institute of Climate-Smart Agriculture, Bundesallee 50, 38116 Braunschweig, Germany

^8^Biodiversity Research Institute, Jeju Technopark, 338 Shinyedong-ro, Jeju 63608, Republic of Korea

^9^Department of Microbiology, Chungbuk National University, 1 Chungdae-ro, Seowon-Gu, Cheongju 28644, South Korea

^*^To whom correspondence should be addressed.

E-mail: [myjung@jejunu.ac.kr](mailto:myjung@jejunu.ac.kr)., chris.j.sedlacek@gmail.com

This file includes:

Supplementary Experimental Procedures

Supplementary Results and Discussion

Supplementary Tables S1 to S2

Supplementary Figure Legends

Supplementary Video 1 to Video 3

Supplementary Figures S1 to S6

Supplementary References

**Supplementary Experimental Procedures**

***^18^O-H_2_O labeling* experiments**

The details of the analysis of isotopic signatures are described elsewhere (1). Briefly, to estimate whether the produced N_2_O derives mainly from nitrification (hydroxylamine) or nitrifier denitrification, N_2_O was analyzed by isotope ratio mass spectrometry (IRMS). The isotopomer signatures of N_2_O were determined as described before by Lewicka-Szczebak *et al.* (2). The δ^18^O of N_2_O (δ^18^O-N_2_O) after cryo-focusing by IRMS using a Delta V IRMS (Thermo-Fisher, Bremen, Germany) allowed simultaneous detection of *m/z* 44, 45, and 46 of the intact N_2_O^+^ as well as *m/z* 30 and 31 for NO^+^ fragment ions (3).

Batch cultures of strains MY2 and MY3 were grown in a range of ^18^O-labeled H_2_O in AFM to determine the oxygen atom incorporation from water into N_2_O. For this ^18^O-labeling experiment, treatments were established with pH values of 6.0 and 7.5 for strain MY2 and with pH values of 5.5 and 7.5 for strain MY3. To estimate O exchange between water and N_2_O precursors as well as isotopic fractionation during N_2_O production, Snider *et al.* (4) developed an experimental setup where a number of incubations were performed with water - as a potential donor of oxygen in N_2_O – that differed in its ^18^O abundance. Following this approach, three types of media differing in the ^18^O signature of H_2_O were prepared by the addition of 0, 8, and 38 µl of a stock solution enriched in ^18^O-H_2_O (10 at%) (Sigma-Aldrich, Taufkirchen, Germany) to the medium to achieve H_2_O with δ^18^O values of H_2_O (δ^18^O-H_2_O) of approximately -9, 10, and 40‰. At least 4 replicate bottles were included for each ^18^O-H_2_O treatment. To check for the mean ^18^O enrichment of the water used for the treatments, we performed a H_2_O extraction from the medium according to the procedure published by Koeniger *et al.* (5). The δ^18^O values of this extracted water were analyzed using cavity ring-down spectroscopy (model L 1115-I, Picarro, Santa Clara, USA,), which was suitable for analyzing H_2_O either as a liquid or in vapor samples. The concentration of produced N_2_O and the isotopic analysis were conducted as described above. Oxygen isotopic composition of O_2_ in the headspace was estimated to be 23.5‰ (6). In accordance with Snider *et al.* (4), δ^18^O values (‰) of N_2_O produced and H_2_O used in the experiment were recalculated by relating ratios of ^18^O/^16^O of N_2_O or H_2_O versus ratios of ^18^O/^16^O of the precursor O_2_ (δ^18^O-N_2_O [vs. O_2_] or δ^18^O-H_2_O [vs. O_2_], respectively). These calculated values were used to perform a linear regression of δ^18^O-N_2_O [vs. O_2_] versus δ^18^O-H_2_O [vs O_2_]. As described by Snider *et al.* 2009, the slope describes the incorporation of O into N_2_O; thus, O exchange and the intercept resembles the associated O isotope fractionation. ^18^O-H_2_O (‰ vs. ratio of ^18^O-O_2_) was determined in replicates for each treatment by equilibration with calibration against ^18^O-H_2_O standards.

***Biofilm formation test***

The biofilm formation experiment was conducted with a tube method (7). Strain MY3 was inoculated (1 ml) into polystyrene test tubes containing 9 ml of AFM and ammonium chloride as a substrate at the following concentrations: 0, 1, 2, 5, 10, and 20 mM. *Nitrosotenuis chungbukensis* MY2 and *Nitrosomonas europaea* were used as ammonia-oxidizing controls and experiments were performed as done for strain MY3. *Pseudomonas aeruginosa* was used as a heterotrophic bacterium control and was inoculated in the same volume at Tryptic Soy Broth (TSB) in place of AFM. In addition, 0.5 mM pyruvate was added to the *N. chungbukensis* MY2 cultures. All cultures were incubated at 30 ℃ without shaking. After 30 days, the concentrations of ammonia and nitrite in each sample were measured colorimetrically to confirm the activity of the ammonia-oxidizing strains. Tubes were washed twice with phosphate-buffered saline (PBS) and the remaining biofilm was stained with safranin at room temperature for 1 hour. The safranin-stained tubes were rewashed twice with PBS and dried overnight. Acetic acid (200 µl, 30%, v/v) was added to dissolve the safranin-stained biofilm and then incubated at room temperature for 15 minutes. The amount of safranin-stained biofilm was quantified colorimetrically at 570 nm using a SpectraMax i3x Multi-mode Microplate Reader (Molecular Devices, USA).

**Supplementary results and discussion**

***Tracing the oxygen in produced N_2_O***

The identification of NH_2_OH and NO as crucial intermediates in AOA metabolism (8, 9) suggests the existence of several possible pathways for N_2_O production, as both NH_2_OH and NO are probable precursors of N_2_O. It has been demonstrated that NH_2_OH produced by AOM also contributes to the abiotic production of N_2_O along with nitrite during aerobic ammonia oxidation (10). Recently, two separate studies have identified novel pathways for N_2_O production from AOA species. The enzymatic NO_2_^-^ reduction by a cytochrome P450 involved pathway was identified in strain MY3 when cultured under lower pH conditions (1), and a novel NO dismutation pathway was proposed in the marine AOA *Nitrosopumilus maritimus* under anaerobic conditions (11). In addition, the hybrid N_2_O formation pathway has been experimentally demonstrated and is considered the dominant pathway (ammonia-N and nitrite-N equally contribute to N_2_O production) for N_2_O production from AOA species (12-14). Therefore, it is still unclear and controversial which N_2_O production mechanisms are used or are dominant by differing AOA species or under a vary of environmental conditions (12-14).

To verify the previous result that strain MY3 grown under low pH conditions does produce N_2_O, oxygen (O) incorporation into N_2_O with ^18^O-labeled H_2_O was traced. In the first step of conventional nitrification (the oxidation of ammonia to hydroxylamine), the oxygen atom incorporated by the ammonia monooxygenase originates from dissolved O_2_. In the subsequent oxidation of hydroxylamine to nitrite, the oxygen atom incorporated originates from H_2_O (15). Results of our ^18^O-H_2_O labeling experiments demonstrated that the δ^18^O-N_2_O produced by AOA is dependent on labeled δ^18^O-H_2_O (Fig. S2). In strain MY3 grown at pH 7.5, less than half (slope = 0.17 ± 0.01) (*P* < 0.001) of the oxygen atoms in the produced N_2_O originated from H_2_O (Fig. S2A), which is similar to the results previously obtained for marine AOA strains (15, 16). This indicates that N_2_O originated from both NH_2_OH and NO_2_^-^ together. At pH 5.5, approximately 50% of the O atoms in N_2_O originated from ^18^O-H_2_O (slope = 0.51 ± 0.03) (*P* < 0.001) in *N. oleophilus* MY3 (Fig. S2B), indicating that a higher proportion of N_2_O was derived from NO_2_^-^ than under neutral pH conditions. This increase likely reflects a physiological shift caused by the pH-dependent conversion of toxic NH_2_OH to its cationic form, NH_3_OH^+^, at pH 5.5, which might prevent or slow its conversion to N_2_O. In contrast, strain MY2 showed only a slight increase in the slope from 0.31± 0.03 (*P* < 0.002) to 0.36 ± 0.01 (*P* < 0.001) at pH 6.0 compared to at pH 7.5 (Fig. S2C and D). If only hybrid formation is involved in the production of N_2_O during ammonia oxidation (NH_2_OH + NO → N_2_O + H_2_O + H^+^), and NO_2_^-^ (NH_2_OH + NO + 2H_2_O → 2NO_2_^-^) (17), the theoretical incorporation rate of ^18^O in N_2_O should be ~31.3%, which it is for strain MY2. Here, because ~62.5% of the ^18^O incorporated comes from NO_2_^-^.

Oxygen exchange does not occur during the production of NH_2_OH, but abiotic exchange after oxidation to nitrite with ^18^O-H_2_O cannot be ruled out completely (18). However, studies that determined the oxygen atom exchange noted comparatively little observed exchange occurred between NO_2_^-^ and H_2_O in AOB and AOA cultures during ammonia oxidation, and ~50% of the O in NO_2_^-^ originated from ^18^O-H_2_O (15, 19). However, in a recent study (16), the ratio of integrated ^18^O from ^18^O-H_2_O to NO_2_^-^ was experimentally observed to be approximately 63%, which is consistent with the hypothesis that the hybrid formation of N_2_O from NH_2_OH and NO, as we already described above. Subsequently, one NO_2_^-^ molecule may be reduced back to NO, while another O atom from H_2_O may be integrated into the NO_2_^-^ molecule, or intracellular O atom exchange may occur during NO_2_^-^ production by AOA. Together, these results corroborate the previous experimental findings that the strain MY3 produces N_2_O by enzymatic reduction of NO_2_^-^ under acidic conditions (1).

***The effect of ammonia concentration on biofilm formation***

How biofilm formation of strain MY3 was affected by varying initial substrate (ammonium) concentrations was investigated in comparison with an AOB strain (*N. europaea*), another AOA (*N. chungbukensis* MY2), and the non-ammonia-oxidizing heterotrophic bacterium (*Pseudomonas aeruginosa*). The hypothesis was that biofilm formation in strain MY3 would be induced when exposed to high ammonium concentrations as strain MY3 has a hydrophobic cell surface, is a known biofilm former, and exhibits higher ammonia oxidation activity when in flocs or a biofilm (20). In addition, when exposed to toxic chemicals or other environmental stress, archaeal cells tend to form biofilms as a form of protection (21). For all bacteria cultures tested, biofilm formation was identified and quantified in hydrophobic polystyrene tubes.

Of the four tested bacteria, only strain MY3 formed significantly (*P* > 0.001) more biofilm in the presence of high (4 and 10 mM) initial ammonium concentrations. However, at 20 mM ammonia, biofilm formation in MY3 was not significantly enhanced, even though the total amount of ammonia oxidation was similar to that in the 10 mM ammonium treatment (Fig. S5). This may imply that there was enough stress in the 20 mM treatment to reduce biomass production. Biofilm formation of the other AOA strain, *N. chungbukensis* MY2, decreased with increasing ammonia concentrations. In contrast, biofilm production by the AOB *N. europaea* and *P. aeruginosa* was unaffected by the varying ammonium concentrations (Fig. S5).

Previously, it has been observed that biofilm formation of *N. europaea* is enhanced when the growth medium is supplemented with organics or when *N. europaea* is co-cultured with a heterotrophic bacterial partner (22-24). However, without additional organics or heterotrophic partner, biofilms formation was not robust (25, 26). This highlights how different ammonia oxidizers regulate their biofilm formation in response to different environmental signals or cues. Therefore, additional studies on the biofilm formation of strain MY3 with various factors that are considered to affect stress induction of AOA, including the addition of organics and co-culturing with heterotrophic bacterial partners, are of interest for future studies.

**Table S1.** Sequences of oligonucleotide primers used for qPCR analysis of transcription in *N. oleophilus* MY3.

| **Primer** | **Target gene** | **Sequence (5’-3’)** |
| --- | --- | --- |
| 195F_(amt)_modi | ammonium transporter | TGCACCGGACGTTAATGGAA |
| 775R_(amt)_modi | ammonium transporter | CGGTAGGCTTTCCAGTCCTC |
| 519F | 16s rRNA | CAGCMGCCGCGGTAA |
| 727R | 16s rRNA | GCTTTCRTCCCTCACCGT |
| Cren 23F(amoA) | *amoA* | ATGGTCTGGCTWAGACG |
| Cren 616R(amoA) | *amoA* | GCCATCCATCTGTATGTCCA |
| 320-02370-F | methylmalonyl-CoA mutase large subunit | CGCGTTCGACCTCGCTACCCA |
| 540-02370-R | methylmalonyl-CoA mutase large subunit | GTACCGGATAACTTCTCCGG |
| 683-03315-F | 4-hydroxybutyryl-CoA dehydratase | CTATGGAAGACAATCGTGCGA |
| 892-03315-R  rpoB-F  rpoB-R | 4-hydroxybutyryl-CoA dehydratase  RNA polymerase B  RNA polymerase B | GATCAATACATCTCCTAATCCGG  AGAAGCGAGGAATCAACGCA  GCTTCGGCTTAGTGGTGACT |

**Table S2.** Clusters of orthologous groups (COGs) automated classifications of the unique core genome of the five *Nitrosocosmicus* genomes analyzed in this study.

| **Functional category** | | **CDS** | **Percent  (/total unique CDS)** |
| --- | --- | --- | --- |
| ***CELLULAR PROCESSES AND SIGNALING*** | | | |
| D | Cell cycle control, cell division, chromosome partitioning | 1 | 0.35 |
| M | Cell wall/membrane/envelope biogenesis | 4 | 1.41 |
| O | Posttranslational modification, protein turnover, chaperones | 5 | 1.77 |
| T | Signal transduction mechanisms | 3 | 1.06 |
| U | Intracellular trafficking, secretion, and vesicular transport | 1 | 0.35 |
| V | Defense mechanisms | 5 | 1.77 |
| J | Translation, ribosomal structure and biogenesis | 11 | 3.89 |
| K | Transcription | 5 | 1.77 |
| L | Replication, recombination and repair | 7 | 2.47 |
| ***METABOLISM*** | | | |
| C | Energy production and conversion | 10 | 3.53 |
| E | Amino acid transport and metabolism | 14 | 4.95 |
| F | Nucleotide transport and metabolism | 5 | 1.77 |
| G | Carbohydrate transport and metabolism | 9 | 3.18 |
| H | Coenzyme transport and metabolism | 13 | 4.59 |
| I | Lipid transport and metabolism | 5 | 1.77 |
| P | Inorganic ion transport and metabolism | 7 | 2.47 |
| Q | Secondary metabolites biosynthesis, transport and catabolism | 1 | 0.35 |
| ***POORLY CHARACTERIZED*** | | | |
| R | General function prediction only | 12 | 4.24 |
| S | Function unknown | 5 | 1.77 |
|  |  |  |  |
|  | **COG gene** | **123** | **43.46** |
|  | **Total unique gene** | **283** | **100.00** |

**Supplementary Figure Legends**

**Fig. S1.** Pairwise whole genome comparison of 39 AOA species compared in this study based on the (A) average amino acid identity (AAI) and (B) average nucleotide identity (ANI) similarity parameters. AOA are represented by strain name and the values provided are percent identity in both cases.

**Fig. S2.** Relationship between the δ^18^O-N_2_O produced (vertical axis) and the δ^18^O-H_2_O used in the growth medium (horizontal axis). Panels A and B show data from *N. oleophilus* MY3 cultured at pH 7.5 (A) and 5.5 (B). Panels C and D show data from *N. chungbukensis* MY2 cultured at pH 7.5 (C) and 6.1 (D). If all N_2_O were produced solely from NO_2_^-^, the slope would be 0.5 (dashed line), and if N_2_O were produced solely from ammonia oxidation (hydroxylamine), no oxygen atoms from H_2_O would be incorporated into N_2_O and the resulting slope would be zero. The *P*-value of all the slopes was less than 0.001.

**Fig. S3.** Segments of the amino acid sequence alignment of AOA ammonium channel transporter (Amt) family proteins. Sequence alignment of the AOA Amt protein based on the program MAFFT (27, 28). The presented regions correspond to the first three extracellular loops (Loop 2) and transmembrane domains 4 to 6 (M4–M6), as referred to in the literature (29). Three residues constituting the proposed ammonium-binding site are indicated with red arrows (S, D, S). Other residues conserved >50 % are highlighted in default Clustal colors.

**Fig. S4.** Cryo-electron tomography analysis of *Nitrososphaera viennensis* cell surface. (A) A representative XY slice from the electron tomographic slices of *N. viennensis* (Supplementary Video 2). (B) An enlargement of the rectangular in panel A. (C) An XZ slice of the region in panel B. Scale bars equal 200 nm in panels A, and 20 nm in panels B and C

**Fig. S5.** Cryo-electron tomography analysis of *Nitrosocosmicus franklandus* cell surface. (A) A whole-cell image of *Nitrosocosmicus franklandus*. (B) An enlargement of the rectangular in panel A. (C) A representative XY slice from the electron tomographic slices (Supplementary Video 3). (D) Two different XY slices of the rectangle region in panel (C). The black arrows in panels (B) and (D) indicate the hair-like structures on the cell surface, and the white arrows in panels (C) indicate the vesicle-like structures in (pseudo)periplasmic space. Scale bars equal 500 nm in panels A and B, and 100 nm in panels (C) and (D).

**Fig. S6.** The effect of varying initial growth medium ammonium concentrations on biofilm formation. Safranin-dyed biofilm formation was quantified for (A) ammonia oxidizers (AOA strains MY3 and MY2, and the AOB *N. europaea*), and (B) the heterotrophic bacteria, *Pseudomonas aeruginosa*. (C) Final concentrations of nitrite produced by the ammonia-oxidizing strains after 30 days of incubation. Each bar represents the mean value (n=2), and the error bars represent the standard deviation.

**Supplementary Video 1**. XY slices from the tomographic three-dimensional reconstruction of the strain MY3.

**Supplementary Video 2.** XY slices from the tomographic three-dimensional reconstruction of the strain *Nitrososphaera viennensis.*

**Supplementary Video 3.** XY slices from the tomographic three-dimensional reconstruction of the strain *Nitrosocosmicus franklandus.*

**Fig. S1.** Pairwise whole genome comparison of 39 AOA species compared in this study based on the (A) average amino acid identity (AAI) and (B) average nucleotide identity (ANI) similarity parameters.

**Fig. S2.** Relationship between δ^18^O-N_2_O (vertical axis) and δ^18^O-H_2_O used in the growth medium (horizontal axis). Panels A and B show data *N. oleophilus* MY3 cultured at pH 7.5 (A) and 5.5 (B). Panels C and D show data for *N. chungbukensis* MY2 cultured at pH 7.5 (C) and 6.1 (D). If all N_2_O were produced solely from NO_2_^-^, the slope would be 0.5 (dashed line), and if N_2_O were produced solely from ammonia oxidation (hydroxylamine), no oxygen atoms from H_2_O would be incorporated into N_2_O and the resulting slope would be zero. The *P*-value of all the slopes was less than 0.001.

**Fig. S3.** Segments of the amino acid sequence alignment of ammonia-oxidizing archaea (AOA) ammonia channel transporter (Amt) family proteins. Sequence alignment of the AOA Amt protein based on the program MAFFT (27, 28). The presented regions correspond to the first three extracellular loops (Loop 2) and transmembrane domains 4 to 6 (M4–M6), as referred to in the literature (29). Three residues constituting the proposed ammonium-binding site are indicated with red arrows (S, D, S). Other residues conserved >50 % are highlighted in default Clustal colors.

**Fig. S4.** Cryo-electron tomography analysis of *Nitrososphaera viennensis* cell surface. (A) A representative XY slice from the electron tomographic slices of *N. viennensis* (Supplementary video 2). (B) An enlargement of the rectangular in panel (A). (C) An XZ slice of the region in panel (B). Scale bars equal 200 nm in panels A, and 20 nm in panels (B) and (C).

**Fig. S5.** Cryo-electron tomography analysis of *Nitrosocosmicus franklandus* cell surface. (A) A whole-cell image of *Nitrosocosmicus franklandus*. (B) An enlargement of the rectangular in panel A. (C) A representative XY slice from the electron tomographic slices (Supplementary video 3). (D) Two different XY slices of the rectangle region in panel (C). The black arrows in panels (B) and (D) indicate the hair-like structures on the cell surface, and the white arrows in panels (C) indicate the vesicle-like structures in (pseudo)periplasmic space. Scale bars equal 500 nm in panels (A) and (B), and 100 nm in panels (C) and (D).

**Fig. S6.** The effect of varying initial growth medium ammonium concentrations on biofilm formation. Safranin-dyed cells indicating biofilm formation was quantified for (A) ammonia oxidizers (AOA strains MY3 and MY2, and the AOB *Nitrosomonas europaea* ATCC19718), and (B) the heterotrophic bacteria, *Pseudomonas aeruginosa*. (C) Final concentrations of nitrite produced after 30 days of incubation of the ammonia oxidizers shown in (A). Each bar represents the mean value (n=2), and the error bars represent the standard deviation of duplicate experiments.

**Supplementary References**

1. Jung MY, Gwak JH, Rohe L, Giesemann A, Kim JG, Well R, Madsen EL, Herbold CW, Wagner M, Rhee SK. 2019. Indications for enzymatic denitrification to N_2_O at low pH in an ammonia-oxidizing archaeon. ISME J 13:2633-2638.

2. Lewicka-Szczebak D, Augustin J, Giesemann A, Well R. 2017. Quantifying N_2_O reduction to N_2_ based on N_2_O isotopocules – validation with independent methods (helium incubation and ^15^N gas flux method). Biogeosciences 14:711-732.

3. Well R, Flessa H. 2008. Isotope fractionation factors of N_2_O diffusion. Rapid Commun Mass Spectrom 22:2621-8.

4. Snider DM, Schiff SL, Spoelstra J. 2009. ^15^N/^14^N and ^18^O/^16^O stable isotope ratios of nitrous oxide produced during denitrification in temperate forest soils. Geochim Cosmochim Acta 73:877-888.

5. Koeniger P, Marshall JD, Link T, Mulch A. 2011. An inexpensive, fast, and reliable method for vacuum extraction of soil and plant water for stable isotope analyses by mass spectrometry. Rapid Commun Mass Spectrom 25:3041-3048.

6. Kool D, Wrage N, Oenema O, Dolfing J, Van Groenigen J. 2007. Oxygen exchange between (de) nitrification intermediates and H_2_O and its implications for source determination of NO and N_2_O: a review. Rapid Commun Mass Spectrom 21:3569-3578.

7. Kırmusaoğlu S. 2019. The methods for detection of biofilm and screening antibiofilm activity of agents, vol 7.

8. Martens‐Habbena W, Qin W, Horak RE, Urakawa H, Schauer AJ, Moffett JW, Armbrust EV, Ingalls AE, Devol AH, Stahl DA. 2015. The production of nitric oxide by marine ammonia‐oxidizing archaea and inhibition of archaeal ammonia oxidation by a nitric oxide scavenger. Environ Microbiol 17:2261-2274.

9. Vajrala N, Martens-Habbena W, Sayavedra-Soto LA, Schauer A, Bottomley PJ, Stahl DA, Arp DJ. 2013. Hydroxylamine as an intermediate in ammonia oxidation by globally abundant marine archaea. Proc Natl Acad Sci U S A 110:1006-11.

10. Liu S, Han P, Hink L, Prosser JI, Wagner M, Bruggemann N. 2017. Abiotic conversion of extracellular NH_2_OH contributes to N_2_O emission during ammonia oxidation. Environ Sci Technol 51:13122-13132.

11. Kraft B, Jehmlich N, Larsen M, Bristow LA, Könneke M, Thamdrup B, Canfield DE. 2022. Oxygen and nitrogen production by an ammonia-oxidizing archaeon. Science 375:97-100.

12. Stein LY. 2019. Insights into the physiology of ammonia-oxidizing microorganisms. Curr Opin Chem Biol 49:9-15.

13. Wu L, Chen X, Wei W, Liu Y, Wang D, Ni B-J. 2020. A critical review on nitrous oxide production by ammonia-oxidizing archaea. Environ Sci Technol 54:9175-9190.

14. Prosser JI, Hink L, Gubry-Rangin C, Nicol GW. 2020. Nitrous oxide production by ammonia oxidizers: Physiological diversity, niche differentiation and potential mitigation strategies. Glob Chang Biol 26:103-118.

15. Santoro AE, Buchwald C, McIlvin MR, Casciotti KL. 2011. Isotopic signature of N_2_O produced by marine ammonia-oxidizing archaea. Science 333:1282-5.

16. Wan XS, Hou L, Kao S-J, Zhang Y, Sheng H-X, Shen H, Tong S, Qin W, Ward BB. 2023. Pathways of N_2_O production by marine ammonia-oxidizing archaea determined from dual-isotope labeling. Proc Natl Acad Sci U S A 120:e2220697120.

17. Kozlowski JA, Stieglmeier M, Schleper C, Klotz MG, Stein LY. 2016. Pathways and key intermediates required for obligate aerobic ammonia-dependent chemolithotrophy in bacteria and Thaumarchaeota. ISME J 10:1836-1845.

18. Casciotti KL, Böhlke JK, McIlvin MR, Mroczkowski SJ, Hannon JE. 2007. Oxygen isotopes in nitrite: Analysis, calibration, and equilibration. Anal Chem 79:2427-2436.

19. Frame CH, Casciotti K. 2010. Biogeochemical controls and isotopic signatures of nitrous oxide production by a marine ammonia-oxidizing bacterium. Biogeosciences 7:2695-2709.

20. Jung MY, Kim JG, Sinninghe Damste JS, Rijpstra WI, Madsen EL, Kim SJ, Hong H, Si OJ, Kerou M, Schleper C, Rhee SK. 2016. A hydrophobic ammonia-oxidizing archaeon of the Nitrosocosmicus clade isolated from coal tar-contaminated sediment. Environ Microbiol Rep 8:983-992.

21. Megaw J, Gilmore BF. 2017. Archaeal persisters: persister cell formation as a stress response in *Haloferax volcanii*. Front Microbiol 8.

22. Ren D, Madsen JS, Sørensen SJ, Burmølle M. 2015. High prevalence of biofilm synergy among bacterial soil isolates in cocultures indicates bacterial interspecific cooperation. ISME J 9:81-89.

23. Lee KWK, Periasamy S, Mukherjee M, Xie C, Kjelleberg S, Rice SA. 2014. Biofilm development and enhanced stress resistance of a model, mixed-species community biofilm. ISME J 8:894-907.

24. Keshvardoust P, Huron VA, Clemson M, Constancias F, Barraud N, Rice SA. 2019. Biofilm formation inhibition and dispersal of multi-species communities containing ammonia-oxidising bacteria. npj Biofilms Microbiomes 5:22.

25. Lauchnor EG, Radniecki TS, Semprini L. 2011. Inhibition and gene expression of *Nitrosomonas europaea* biofilms exposed to phenol and toluene. Biotechnol Bioeng 108:750-757.

26. Powell S, Prosser J. 1992. Inhibition of biofilm populations of *Nitrosomonas europaea*. Microb Ecol:43-50.

27. Katoh K, Rozewicki J, Yamada KD. 2019. MAFFT online service: multiple sequence alignment, interactive sequence choice and visualization. Briefings in bioinformatics 20:1160-1166.

28. Kuraku S, Zmasek CM, Nishimura O, Katoh K. 2013. aLeaves facilitates on-demand exploration of metazoan gene family trees on MAFFT sequence alignment server with enhanced interactivity. Nucleic acids research 41:W22-W28.

29. Offre P, Kerou M, Spang A, Schleper C. 2014. Variability of the transporter gene complement in ammonia-oxidizing archaea. Trends Microbiol 22:665-75.
